# Luteolin: a flavonoid with a multifaceted anticancer potential

**DOI:** 10.1186/s12935-022-02808-3

**Published:** 2022-12-08

**Authors:** Parteek Prasher, Mousmee Sharma, Sachin Kumar Singh, Monica Gulati, Dinesh Kumar Chellappan, Flavia Zacconi, Gabriele De Rubis, Gaurav Gupta, Javad Sharifi-Rad, William C. Cho, Kamal Dua

**Affiliations:** 1 Department of Chemistry, University of Petroleum & Energy Studies, Dehradun, 248007 India; 2 Department of Chemistry, Uttaranchal University, Dehradun, 248007 India; 3 School of Pharmacy and Pharmaceutical Science, Lovely Professional University, Phagwara, India; 4 Discipline of Pharmacy, Graduate School of Health, University of Technology Sydney, Sydney, NSW 2007 Australia; 5 Department of Life Sciences, School of Pharmacy, International Medical University, Bukit Jalil, 57000 Kuala Lumpur, Malaysia; 6 Departamento de Quimica Orgánica, Facultad de Química y de Farmacia, Pontificia Universidad Católica de Chile, Av. Vicuna Mackenna 4860, Macul, 7820436 Santiago, Chile; 7 Institute for Biological and Medical Engineering, Schools of Engineering, Medicine and Biological Sciences, Pontificia Universidad Católica de Chile, 7820436 Santiago, Chile; 8 Faculty of Health, Australian Research Centre in Complementary and Integrative Medicine, University of Technology Sydney, Ultimo, Australia; 9 School of Pharmacy, Suresh Gyan Vihar University, Jaipur, Rajasthan India; 10 Facultad de Medicina, Universidad del Azuay, Cuenca, Ecuador; 11 Department of Clinical Oncology, Queen Elizabeth Hospital, Kowloon, Hong Kong China; 12 Department of Pharmacology, Saveetha Dental College, Saveetha Institute of Medical and Technical Sciences, Saveetha University, Chennai, India; 13 Uttaranchal Institute of Pharmaceutical Sciences, Uttaranchal University, Dehradun, India

## Abstract

Therapeutic effect of phytochemicals has been emphasized in the traditional medicine owing to the presence of bioactive molecules, such as polyphenols. Luteolin is a flavone belonging to the flavonoid class of polyphenolic phytochemicals with healing effect on hypertension, inflammatory disorders, and cancer due to its action as pro-oxidants and antioxidants. The anticancer profile of luteolin is of interest due to the toxic effect of contemporary chemotherapy paradigm, leading to the pressing need for the development and identification of physiologically benevolent anticancer agents and molecules. Luteolin exerts anticancer activity by downregulation of key regulatory pathways associated with oncogenesis, in addition to the induction of oxidative stress, cell cycle arrest, upregulation of apoptotic genes, and inhibition of cell proliferation and angiogenesis in cancer cells. In this review, we discuss about the anticancer profile of luteolin.

## Introduction

Cancer claims a towering morbidity and mortality rate worldwide. As per the report published by World Health Organization on 3 February 2022, cancer is the leading cause of death around the globe with 10 million total deaths alone in the year 2020 due to lung cancer (1.80 million deaths of 2.21 million cases), colon cancer (0.91 million deaths of 1.93 million cases), and breast cancer (0.68 million deaths of 2.26 million cases) (https://www.who.int/news-room/fact-sheets/detail/cancer). Chemotherapy has served as a silver lining to the cancer patients; however, the side effects and a sizeable mortality rate demands alternative treatment regime with physiologically safe drugs to which the naturally occurring compounds present a robust candidature. Furthermore, the evolution of drug resistance in cancer cells aggravates the need for safer alternatives with optimal efficacy for sensitizing the cancer cells towards conventional drugs [1].

Phytochemicals offer a resolute anticancer profile by providing novel pharmacophores and bioactive molecules that not only inhibit the development and progression of cancers by modulating the key regulatory pathways but ameliorate the chemo-sensitization of cancer cells. In addition to this, anticancer phytochemicals simultaneously target diverse, interconnected oncological pathways that further prevent the relapse and evade the resistance mechanisms in oncologic cells [2]. Of the diverse nature of anti-oncologic phytochemicals, the phenolics present a commendable profile with multifaceted modes of action towards cancer-associated pathways.

**Fig. 1:**
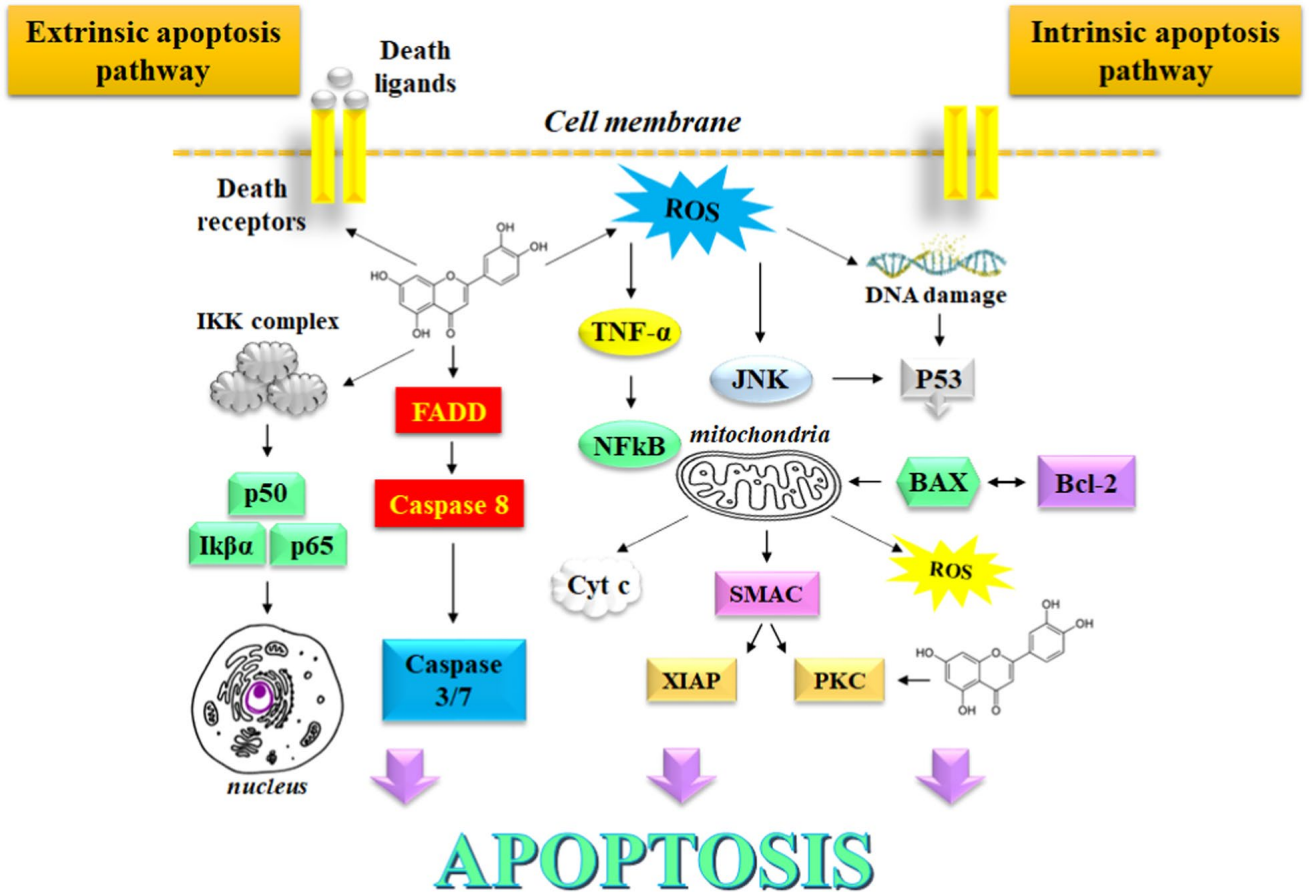
Signaling pathways affected by luteolin

Luteolin is a phenolic phytochemical belonging to the flavone class of flavonoids with a notable therapeutic potential which has been utilized in the management of carcinogenesis by the induction of apoptosis, activation of cell cycle arrest, mitigation of angiogenesis, metastasis, and cell proliferation [3]. The anticancer effects of Luteolin have been observed in malignancies where it effectively modulates the expression of multifaceted oncological pathways that work in tandem to manifest tumorigenesis. Luteolin induces mitochondrial dysfunction and activates the endoplasmic reticulum stress response in glioblastoma cells, which triggers the generation of intracellular reactive oxygen species (ROS) [4]. These events further activate the expression of stress-related proteins by mediating the phosphorylation of PERK, ATF4, eIF2α, and cleaved-caspase 12. Luteolin is known to reverse epithelial-to-mesenchymal transition (EMT), which is associated with the cancer cell progression and metastasis. These events are caused by the dwindling of cytoskeleton and by upregulating the biomarker E-cadherin expression, followed by a significant downregulation of the N-cadherin and vimentin expression [5]. Furthermore, luteolin holds potential to improve the spinal damage and brain trauma caused by 1-methyl-4-phenylpyridinium due to its excellent neuroprotective properties. Luteolin-mediated sensitization of cancer cells ameliorates the chemotherapy-induced cytotoxicity due to the downregulation and suppression of cellular pathways such as nuclear factor kappa B (NF-kB), phosphatidylinositol 3’-kinase (PI3K)/Akt, and X-linked inhibitor of apoptosis protein (XIAP) [6]. Owing to a remarkable anticancer profile, luteolin serves as an attractive molecule for the development of impending anticancer drugs. In this review, we discus about the robust anticancer profile of luteolin. The signaling pathways affected by luteolin are depicted in  Fig. 1.

### Luteolin as redox stress regulator

Onset of redox stress resulting in the cascade of several deleterious biochemical and physiological events marks the commencement of oncogenesis. It mainly arises from the generation of a diverse group of highly reactive, oxygen containing species with a short lifespan that serve as second messengers for cellular signaling. These include hydrogen peroxide (H_2_O_2_), hydroxyl radical (.OH), singlet oxygen (^1^O_2_), superoxide radical (O_2_^-^), and lipid peroxyl radical (LOO). The reactive oxygen species (ROS) are known to manifest irreparable damage to DNA, proteins, and lipids. that eventually causes diseases including cancer.

The regulation of redox stress by luteolin plays an important role in exerting the anticancer activity, by exerting the antioxidant effect arising due to the oxidation of its phenolic groups where the hydrogen atom may be donated to the free radicals. Notably, luteolin exhibits pro-oxidant properties that support the onset of apoptosis in tumor cells due to ROS-mediated damage to nucleotides and proteins, which also interferes with cellular signaling by ROS production that plays an important role in triggering apoptosis. The main pathways involved in this event include suppression of NF-kB pathway, and activation of JNK pathway that induces TNF-mediated cytotoxicity to cancer cells.

Kang et al. [7] reported luteolin-induced redox stress for the induction of apoptotic cell death in human colon cancer cells. The onset of apoptosis occurs due to the activation of mitochondria-mediated caspase pathway in HT-29 cells. Furthermore, the membrane action potential of mitochondria depletes in the presence of luteolin, Ca^2+^ levels and Bax expression upregulate, the levels of caspase-3 and caspase-9 increase, while the downregulation of Bcl-2 expression occurs. These events cause the cytosolic release of cytochrome c from mitochondria that onsets the production of intracellular and mitochondrial ROS scavenging antioxidant enzymes such as catalase and dismutase.

Luteolin is further known to raise the level of reduced glutathione and upregulated the expression of glutathione synthetase which is a potent antioxidant [8] (Furthermore, it has been observed that luteolin induces apoptosis in human colon cancer cells by activating mitogen-activated protein kinase signaling pathway that plays an important role in programmed cell death [9]. In vivo experiments on animal models with diethylnitrosamine-induced liver cancer indicated amelioration in the levels of enzymatic oxidants such as catalase, superoxide dismutase; marker enzymes such as alanine/ aspartate transaminases; α-fetoprotein; and accumulation of lipid peroxides in liver tissues and plasma [10].

Besides, several metals and metal ions are known to trigger a series of events that cater to the production of ROS and generate oxidative stress in cells. Free radical scavenging by metal chelation serves as an attractive approach for exerting the antioxidant effect. Especially, the low molecular weight metal chelators have received a considerable attention as therapeutics that function by mitigating the oxidative stress generated by metal ions by scavenging the free radicals generated thereof. Luteolin serves as a good metal-chelating agent owing to the presence of dihydroxyl substituents on the aromatic ring framework. These groups can chelate a variety of divalent, trivalent and tetravalent metal ions including Zn(II), V(II), Cu(II), Fe(II), Sn(II), Fe(III), Cr(III), Vn(IV), and Mo(IV) [11]. Recently, the complexation of Mn(II) with luteolin has been reported through the carbonyl group and the adjacent hydroxyl group of latter [12]. This complex has been reported to show enhanced antioxidant activity as compared to the parent molecule itself. Luteolin-Mn(II) complex reversibly inhibited the xanthine oxidase in competitive manner, which is a main enzyme involved in the production of reactive oxygen species [13].

Upon exposure to different ultraviolet (UV-A) radiations, the human skin fibroblasts exhibit autophagy and a burst release of ROS. Incubation with micromolar concentration of luteolin is known to protect human skin fibroblasts from ultraviolet (UV-A) radiation by decreased autophagy as ascertained by fluorescence and transmission electron microscopy. The ROS levels also lowered as indicated by flow cytometry. Furthermore, the treatment with luteolin led to downregulation of the expression of hypoxia-inducible factor-1α and autophagy-associated proteins, Beclin 1, and LC3 in UV-A irradiated human skin fibroblasts as indicated by western blotting assay [14].

The protective effect of luteolin against the photoaging effect of UV-B radiation is noteworthy. The irradiation of human dermal fibroblasts with UV-B led to raised ROS levels in the skin and tissues causing a significant damage to the skin of animal models. As observed in the hematoxylin–eosin and Masson staining. The detection of ROS levels was done with the help of dihydroethidium (DHE) and dichlorofluorescein (DCF) fluorescent probes. Luteolin reportedly relieved UV-B induced wrinkle formation and erythema and improved cell viability. Furthermore, luteolin lowered the levels of oxidative stress, and downregulated the activation of matrix metalloproteinases (MMPs). Luteolin provides protection to the ageing skin cells under the influence of UV-B radiation by targeting SIRT3/ROS/MAPK pathways (Mu et al. 2021).

Luteolin exhibits pro-oxidant potential that plays a critical role in inducing apoptosis in cancer cells. Ju et al. [15] provided evidence on the luteolin-induced accumulation of ROS that modulates the expression of TNF-activated pathways in lung cancer cells. The expression of NF-kB is downregulated, while the expression of c-Jun N-terminal kinase is potentiated that instigates apoptosis in lung cancer cells induced by tumor necrosis factor. Moreover, luteolin further triggered an early phase accumulation of ROS due to the suppression of the activity of cellular superoxide dismutase.

Luteolin extracted from the leaves of *Clerodendrum cryptophyllum* Turcz exerts antioxidant activity and negligible cytotoxicity or genotoxicity in TBHP (tert-Butyl hydroperoxide)-sensitized HepG2 cells. Preincubation of cells with 1–40 µM concentration of Luteolin followed by their direct exposure to TBHP significantly improved the cell viability in a dose dependent manner. Luteolin reportedly demonstrated an optimal 43.7% inhibition of the accumulation of ROS, 24.5% decrease in malondialdehyde levels, and 38.7% lowering of lactate dehydrogenase levels at a concentration of 30 µM. Similarly, the expression of superoxide dismutase ameliorated by 73.7%, while the activity of glutathione improved by 72.3% at the same concentration of luteolin. The results from western blot assay showed an upregulation of procaspase-3 and a downregulation of cleaved caspase-3 that eventually resulted in the countering of TBHP-instigated oxidative stress [16].

Oxidative stress has been found to be a major contributing factor towards the progression of neurodegeneration and ensuing disorders such as Alzheimer’s disease and Huntington’s disease. Luteolin has been reported to exert antioxidant effect and neuroprotective effect in sodium nitroprusside-sensitized animals. The coadministration of 3–30 nM luteolin and 10 nM sodium nitroprusside in corpus striatum provides defense against ROS-mediated motor dysfunction and brain damage in animals by Fe^2+^ induced lipid peroxidation in homogenate of mouse brain.

The similar effect is achieved for 600–1200 mg/kg of orally administered luteolin. Encouraging results obtained from ferrozine assay validated that luteolin at a concentration of 30–100 µg/ml shows Fe^2+^ chelating property and potent DPPH radical scavenging ability at 1–100 µg/ml concentration. These inferences indicated that administration of luteolin by oral or intrastriatal routes provides defense to the mice brain against sodium nitroprusside-induced oxidative damage due to the free radical scavenging and chelating effect of luteolin [17].

Poor bioavailability of luteolin limits its optimal therapeutic efficacy and bioactivity. In order to improve these parameters, mono-acylated derivatives of luteolin have been developed to display radical scavenging activity in addition to showing antiproliferative activity against MDA-MB-231 breast cancer cell lines and HCT116 colon cancer cell lines. The acylation of hydroxyl groups improves the lipophilicity of the test compounds that improves their bioavailability. Furthermore, the radical scavenging property of these derivatives against 2,2′-azino-bis(3-ethylbenzothiazoline-6-sulfonic acid) (ABTS) radical cation and 2,2-diphenyl-1-picrylhydrazyl (DPPH) radical was retained as that of the parent luteolin molecule while keeping Trolox as standard [18].

### Luteolin as inhibitor of cell cycle progression

Cyclin-dependent kinases (CDK)-cyclin complexes regulate the cell cycle responsible for cell growth, development, and differentiation. The dysregulation of cell cycle transforms normal cells to tumor cells. Considering its association towards the development of tumorigenesis, the chemical agents that arrest cell cycle progression have drawn a considerable attention towards the development of anticancer therapeutics [19].

Treatment of MDA-MB-231 cells with luteolin led to dose dependent arrest of cell cycle in S phase by reducing the levels of telomerase and by inhibiting the phosphorylation of NF-kB inhibitor α along with its target gene c-Myc. These events led to the suppression of the expression of human telomerase reverse transcriptase (hTERT) encoding for the catalytic subunit of telomerase. Also, luteolin suppressed the development of breast cancer cells and induced apoptosis to check their progression [20].

Park et al. [21] provided evidence for the arrest of cell cycle by luteolin by studying its anticancer effect on MCF-7 breast cancer cells. Incubation of cancer cells with luteolin caused morphological changes in nucleus and caused perturbation in the progression of cell cycle at sub-G1 and G1 phases in MCF-7 cells. Moreover, luteolin upregulated the expression of caspase cascades and death receptors, including DR5. Luteolin also augmented the activity of caspase-3/-8/-9 in a dose dependent manner and inactivated poly-ADP ribose polymerase (PARP), which is a prime factor that assists the cancer cells to repair themselves. Furthermore, luteolin triggered collapse of mitochondrial membrane potential followed by the release of cytochrome* c*. Eventually, Bax expression augments in the presence of luteolin, while the expression of Bcl-2 is inhibited. Overall, these results suggest the arresting of cell cycle and induction of apoptosis in cancer cells incubated with luteolin.

Treatment of HeLa cells with luteolin has been shown to exhibit cytotoxicity in a dose- and time-dependent manner. Luteolin also exhibited anti-proliferative activity as indicated by the accumulation of luteolin-incubated cells in sub-G1 phase of cell cycle. Treatment with luteolin causes depolarization of mitochondrial membrane potential that manifests cytotoxicity towards cancer cells. Furthermore, the expression of proapoptotic genes such as FAS, FADD, BAX, BAD, BOK, BID, TRADD upregulates, while the anti-apoptotic genes NAIP, BCL-2, and MCL-1 experience downregulation. The expression of cell cycle regulatory genes CDK2, CDKN2B, CCNE2, CDKN1A, and CDK4 decreased on incubation with luteolin.

At transcriptional level, the expression of MAPK1, MAPK3, MAP3K5, MAPK14, PIK3C2A, PIK3C2B, AKT1, AKT2, and ELK1 downregulated. Notably, the expression of PRAS 40 (p-Ther246), PDK1(pser241), PTEN (p-ser380), GSK3b (pser9), AKT (pser473), BAD (pser112), RISK2 (pser386), P70S6k (pThr421/ser424), ERK1 (pT202/Y204), ERK2 (pY185/Y187), and MTOR (pser2448) downregulated in the presence of luteolin in a dose-dependent manner. Overall, these results suggested the display of anti-proliferative and apoptosis triggering properties of luteolin due to the inhibition of MAPK and AKT pathways and due to the arresting of cell cycle by luteolin [22].

The mechanism of inhibition of cell cycle and pro-apoptotic effect of luteolin on human non-small-cell lung carcinoma cell lines A549 was analyzed by Cai et al. [23]. The flow cytometry investigations have revealed increased number of apoptotic cells and G2 phase cells indicating the non-progression of cells beyond G2 phase. The western blot assay indicated the activation of JNK by luteolin, augmented the production of Bax, and increased cleavage of procaspase-9 and caspase-3. The pretreatment of A549 cells with luteolin led to an inhibition of TNFα-mediated trans-nuclear translocation of NF-kB.

Luteolin is also found to arrest cell cycle in G2/M phase in addition to the induction of apoptosis in human colon cancer cells xenografts. The flow cytometric analysis has validated the observation that luteolin promotes cell cycle arrest in G2/M phase and induces apoptosis in LoVo human colon cancer cells in time- and dose-dependent manner. Similar observation is obtained with western blot analysis which revealed that luteolin posed inhibitory effect on cell proliferation on LoVo cells by arresting cell cycle at G2/M phase transition and inactivated cyclin B1 and cell division cycle 2. These events are mediated by cytochrome c and deoxyadenosine triphosphate-mediated activation of apoptotic protease activating factor 1. Further in vivo analysis suggested a decrease in the body weight of colon tumor mice. Overall, these findings indicated chemopreventive and chemotherapeutic effect of luteolin against human colon cancer [24].

Luteolin has been reported to exert cytotoxicity against human hepatocellular carcinoma cell lines HepG2, PLC/PRF/5, HA22T/VGH, and SK-Hep-1. These cell lines on treatment with luteolin demonstrate typical changes in apoptosis with specific DNA laddering pattern. Luteolin reportedly activated caspase-3, and aggravated Bax protein with a simultaneous lowering of Bcl-X_L_ levels. This evidence supports the induction of apoptosis in selected cancer cell lines. Luteolin also mediated arrest of cell cycle in Go/G1 phase that results in anticancer effect [25].

Similar observation has been reported on human breast cancer cell line MDA-MB-453 via regulation of cell cycle and induction of apoptosis and antiproliferative effect. Treatment of these cancer cell lines with luteolin at various doses led to a remarkable lowering of cancer cell growth in a time dependent manner. Notably, the incubation of cancer cells with luteolin results in a considerable decrease in the population of sub-G1 phase cells thereby indicating the arrest of cell cycle in this phase [26]. Luteolin has been investigated to show cytotoxicity activity on human immortalized keratinocytes (HaCaT), and human melanoma cells (A375). Luteolin exerts cell cycle arrest and apoptosis in these cell lines as indicated in the flow cytometry and cellular DNA fragmentation assay. Incubation of the test cancer cell lines with luteolin for 24 h led to accumulation of cells in G2/M phase for HaCaT cells and in G0/G1 phase for A375 cells. The inhibition of cell proliferation, induction of apoptosis, and triggering of cell cycle arrest by luteolin is responsible for the programmed cell death of skin cancer cells [27].

### Luteolin-induced autophagy

Autophagy has been identified as a potential approach for limiting the proliferation and viability of cancer cells. Autophagy is a catabolic process that provides energy during starvation. This energy is derived from the degradation of aged cells and organelles, in addition to the toxic cellular components. Autophagy begins with the disruption of mitochondria and various organelles in cytoplasm. The main role of autophagy is to maintain homeostasis of eukaryotic cells during abiotic and biotic stress. The cellular mechanism of autophagy includes the transferring of damaged cells and organelles and deleterious proteins into lysosomes for the onset of their degradation. The simple end products of degradation, such as amino acids are eventually recycled in the cells.

Owing to its role in maintaining normal functioning and metabolism of healthy cells, autophagy has been identified as a potential approach in the contemporary anticancer therapy. Autophagy plays a dual role of tumor suppression, in addition to its promotion and proliferation depending on the regulation of oncogenic factors such as mTOR, AMPK, PI3K, and AKT. The optimal operation of autophagy plays an important role for the suppression of tumor by reducing the damaged cellular components for upholding the cellular homeostasis.

Reportedly, the autophagy related gene Beclin 1 (BECN1), which plays an important role in the formation of phagophore and tumor suppression, is found to deplete in the human ovarian, breast, prostate cancers, as well as hepatocellular carcinoma and squamous-cell carcinomas. The same has been ascertained in tumor animal models and cancer-cell lines where a downregulation of BECN1 manifests a marked reduction in autophagy followed by enhanced cell proliferation.

Luteolin is known to regulate the expression of Beclin 1, which is an important regulator of autophagy during the nucleation step that results in tumor suppression. Luteolin is also known to regulate the expression of immunoglobulin proteins that bind to endoplasmic reticulum chaperone, and to activate the endoplasmic reticulum stress sensors which includes phosphorylation of eukaryotic initiation factor 2α and splicing of Xbox-binding protein 1 mRNA. Similarly, luteolin exerts autophagy at the elongation step by downregulation of LC3, which is a marker of autophagosomes and found to express in gastrointestinal cancers.

P53 signaling is closely related with the induction or suppression of autophagy thereby presenting a desirable approach towards the anticancer therapy. Functioning as a nuclear transcription factor and mediating the transactivation of proapoptotic, cell cycle arresting proautophagic genes, p53 is considered as a master regulator of autophagy depending on its subcellular localization. Yoo et al. [28] reported the anticancer effect of luteolin due to the induction of apoptosis and autophagy in colon cancer cells. In the presence of luteolin, the HCT116 cells showed heightened phosphorylation of p53 and p53 target gene expression eventually resulting in apoptosis and cell cycle arrest. Autophagy is induced by luteolin in p53 wild-type cells and not in mutant p53 cells. This evidence suggests that autophagy by luteolin is p53 dependent.

Evidence for the induction of apoptosis and autophagy in human hepatocellular cancer Hep3B cells was provided by Lee et al. [29]. To study the effect of luteolin on autophagy, the protein levels of LC3-II and p62 which are considered as markers of autophagy was measured. The levels of LC-II increase on the formation of autophagosomal membranes due to the conjugation of phosphatidylethanolamine with cytosolic LC3. The fusion of lysosomes with autophagosome leads to the degradation of receptor protein p62 along with the LC3-II proteins lying inside and outside of the autophagosome. Therefore, the level of p62 is reduced with a simultaneous increase in the LC3-II levels during autophagy.

Similarly, the protein levels of p62 downregulated while that of LC3-II is upregulated following the luteolin treatment in p53 wild type HepG2 cells indicating the induction of autophagy. This evidence was further validated by analyzing the number of viable cells in the presence of luteolin and autophagy inhibitors 3-methyladenine and chloroquine where the number of viable cells considerably lowered. Luteolin-induced autophagy in cancer cells was further validated by Lee et al. [30]. The A172 and U-373MG cells treated with luteolin showed reduced viability in concentration- and dose-dependent manner. At a concentration above 100 µM, the nucleus of these cells undergoes fragmentation and characteristic morphological changes start to appear that confirm the onset of apoptosis. Notably, the fragmentation of apoptosis-related factors caspase-3, and poly(ADP-ribose) polymerase (PARP) was observed that further confirms the onset of apoptosis at 50 µM concentration.

Luteolin treatment reportedly increased the number of intracellular autophagosomes, as indicated by an increased expression of Beclin 1, and conversion of LC3B-I to LC3B-II in hepatocellular carcinoma SMMC-7721 cells. These events suggest the onset of autophagy in cancer cells followed by their exposure to luteolin which was validated by a reduction in luteolin-induced autophagy in the presence of autophagy inhibitor chloroquine. However, the effect of autophagy was only partial as cell cycle arrest and induction of apoptosis were also noticed as contributing factors [31].

Luteolin has been reported to sensitize the human liver cancer cells towards autophagy induced by tumor necrosis factorrelated apoptosisinducing ligand (TRAIL), which is a type II transmembrane protein of the TNF superfamily. TRAIL induces apoptosis in cancer cells without harming the healthy cells that make it a desirable target for the treatment of various cancer types. However, some cancers develop resistance towards TRAIL-mediated apoptosis which necessitate their sensitization. Treatment of TRAIL-resistant Huh7 cells with luteolin and TRAIL results in synergistic effect on cells.

Reportedly, TRAIL prompts the formation of autophagosomes and its effect on promoting autophagy is determined by the expression of autophagy related genes (ATGs) including Atg5, Atg7, and beclin-1 however, the silencing of JNK pathway results in the attenuation of TRAIL-induced expression of ATGs. In vivo investigations on A549 cell lines established TRAIL-induced autophagy by modulation of ATG expression via JNK pathway that further causes apoptosis and inhibition of cell proliferation. Similarly, luteolin induces autophagic flux in the liver cancer cells that is lowered after the application of autophagy inhibitors such as chloroquine.

The treatment of cancer cells with genetically modified autophagy-related 5 siRNA caused a marked reduction in luteolin-triggered sensitization effect on TRAIL. Pre-treatment of cells with c-Jun N-terminal kinase (JNK) inhibitor SP600125 caused a remarkable inhibition of luteolin-instigated surge of the expression of DR5, which suggests the relation between promotion of DR5 expression by JNK activation [32].

**Fig. 2:**
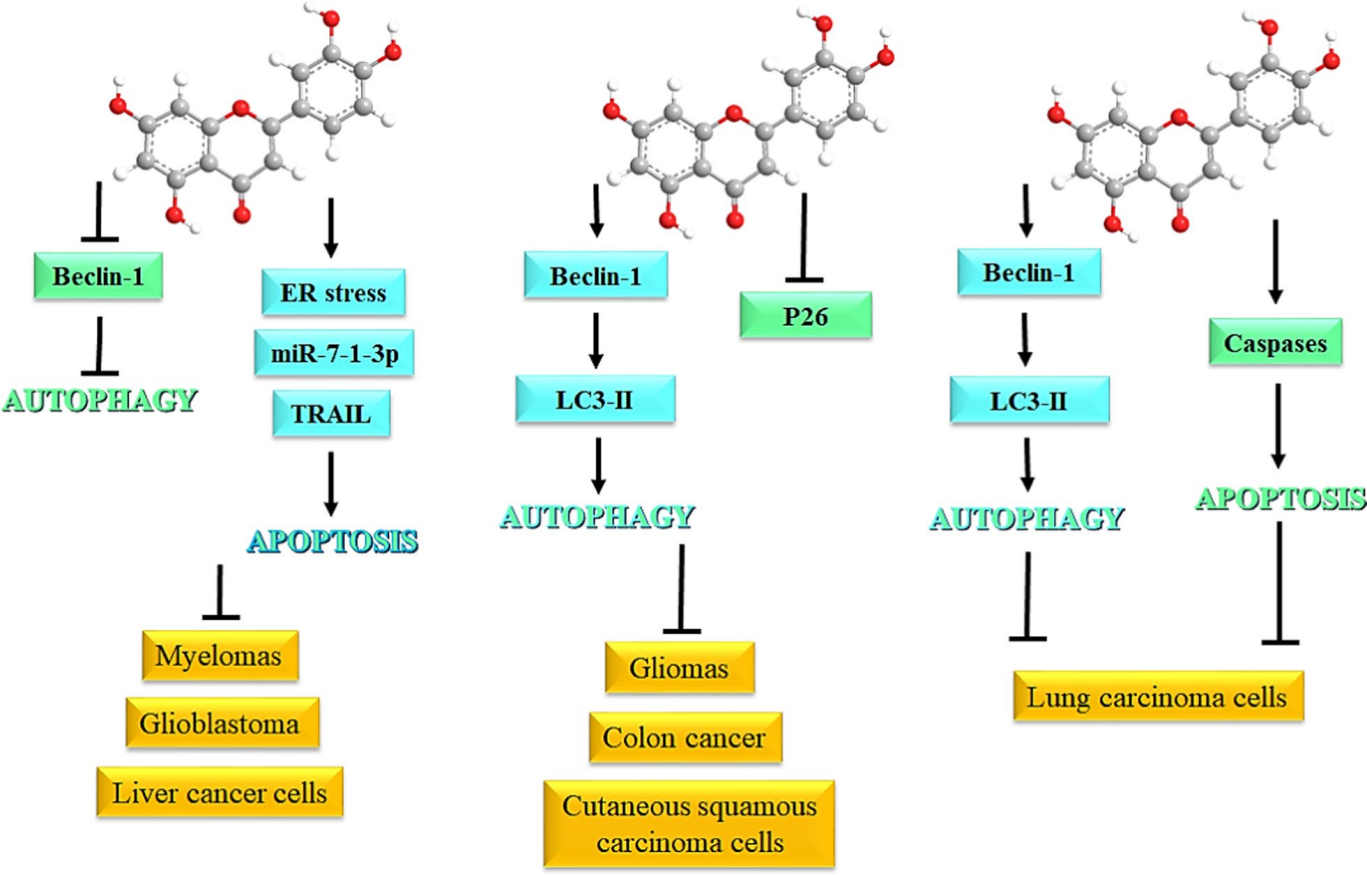
Regulation of autophagy and apoptosis by luteolin

Bcl-2 presents a molecular link between apoptosis and autophagy and its downregulation is known to activate the expression of Beclin-1, which is key regulator of apoptosis and autophagy. Luteolin is also reported to induce apoptosis and autophagy in mouse macrophage ANA-1 cells through Bcl-2 pathway. Incubation of cells with luteolin lowered their viability and led to the downregulation of Bcl-2 expression, induced autophagy and apoptosis, while upregulating the expression of caspase-3/-8. Furthermore, the levels of ATG12, ATG7, and LC3-1 increased while suppression in the levels of Beclin-1 occurs. Treatment with luteolin activated p38, Akt, and JNK signaling pathways that play an important role in autophagy and apoptosis. The pathways through which luteolin regulates autophagy and apoptosis are depicted in Fig. 2.

### Luteolin in the management of various types of cancers

The effects of the Luteolin in various types of cancers summarized in the Table 1.

**Table 1:** Effects of the luteolin in various types of cancers

Colon cancer		
Cancer model	Outcomes	References
Azoxymethane induced colon cancer in Balb/C mouse	Reduction in the expression of iNOX and COX-2	[33]
Azoxymethane induced colon cancer in Balb/C mouse	Increased activity of Glutathione-S-transferase, enhanced expression of Nrf2, activation of GST-α and GST-µ.	[34]
Azoxymethane induced colon cancer in Balb/C mouse	Anti-metastatic activity due to a decrease in MMP-2 and MMP-9	[35]
HT-29 colon adenocarcinoma cell line	Downregulation of PI3K/Akt and ERK1/2 and a reduction in OGF-1R signaling	[36]
Human colon cancer SW620 cells	Inhibition of ERK expression, increase in FOXO3A expression, activation of ERK1/2 and forkhead box O3a signaling pathways, increased expression of autophagic proteins, induction of apoptosis	[37]
Human colon cancer cells HT-29	Apoptotic cell death by upregulating Nrf2 expression via DNA demethylase	[38]
Human colon cancer cells HCT-116	Suppression of NLRP3/IL-1 β signal axis	[39]
Dextran sodium sulfate-induced colitis mice	Downregulation of HMGB1-TLR-NF-κB signaling pathway protein	[40]
Colon cancer DLD-1cells	Downregulation of Notch1 and TGF-β pathways	[41]
Human colorectal cancer HCT116 cells	Epigenetic activation of Nrf2 pathway leading to the inhibition of colorectal carcinogenesis	[42]
Breast cancer		
Breast cancer cell line MCF-7	PLK-1 mediated anticancer activity	[43]
Androgen receptor-positive triple negative breast cancer	Regulation of the expression of MMP9, reduction in the levels of AKT/mTOR-inducing H3K27Ac and H3K56Ac.	[44]
Breast cancer cell lines MDA-MB-453 and MCF-7	Increased expression of miR-203 and the inhibition of Ras/Raf/MEK/ERK signaling.	[45]
Triple negative breast cancer cells MDA-MB-231 and BT5-49	Reversing the epithelial-to-mesenchymal transition via downregulation of the expression of β-catenin	[46]
Triple negative breast cancer cells MDA-MB-231	Induction of apoptosis	[47]
Triple negative breast cancer cells MDA-MB-231 and 4T1	Suppression of epithelial-to-mesenchymal transition expression and Inhibition of YAP/TAZ activity	[48]
Triple negative breast cancer cells MDA-MB-231	Suppression of Notch signaling and miRNA regulation	[49]
Human breast cancer cell lines BT474 and MCF7	Lowering of the methylation and upregulating of the expression of OPCML gene.	[50]
Human breast cancer MDA-MB-435 and MDA-MB-231 (4175) LM2 TNBC cells	Blocking of VEGF production and KDR-mediated activity, inhibition of the migration of tumor cells	[51]
Triple negative breast cancer cells MDA-MB-231	Downregulation of Nrf-2 expression	[52]
Lung cancer		
Human non-small cell lung cancer A549 cells	Suppression of focal adhesion kinase and suppression of non-receptor kinase signaling pathway	[53]
Human non-small cell lung cancer A549 cells	Cell cycle arrest in G2 phase	[23]
Human non-small cell lung cancer A549 cells and H460 cells	Caspase activation induced by Poly(dA:dT), and cleavage of IL-1β in NSCLC cells	[54]
Human non-small cell lung cancer A549 cells	Suppression of inducible PD-L1 expression for ameliorated anticancer activity in KRAS mutant lung cancer	[55]
Human non-small cell lung cancer A549 cells and H460 cells	Induction of apoptosis by the induction of MicroRNA-34a-5p	[56]
Human normal lung epithelial cell NL-20 and lung cancer cell lines NCI-H1975 and NCI-H1650	Downregulation of LIMK1 and its interaction with cofilin	[57]
T790M mutant NSCLC cells	Anti-tumorigenic effect on EGF receptor L858R/T790M mutation and erlotinib-resistant NSCLC	[58]
Human non-small cell lung cancer A549 cells	Cell cycle arrest in G1 phase and apoptosis	[59]
Lung adenocarcinoma A549 cells	Attenuation of TGF-β1-induced epithelial-mesenchymal transition of lung cancer cells	[60]
Lewis lung cancer	lung cancer-induced bone pain by inhibiting NLRP3 inflammasomes and glial activation	[61] e
Prostate cancer		
Human prostate cancer PC-3 cells	Downregulation of calcium-activated chloride channel Anoctamin 1	[62]
Human prostate cancer PC-3 cells	Inhibition of cell-cell adhesion by E-cadherin through AKT/mdm2 pathway	[63]
Human prostate cancer PC-3 cells	Suppression of Wnt signaling by upregulation of FZD6 and suppressing of the stemness of cancer cells	[64]
Human prostate cancer PC-3 cells	Suppression of angiogenesis mediated by Vascular Endothelial Growth Factor Receptor 2 Suppressing	[65]
Human prostate cancer PC-3 and LNCaP cells	Inhibition of cell proliferation via miR-301	[66]
Human prostate cancer PC-3 cells	Induction of miR-630 and inhibition of cyclin G-associated kinase	[67]
Rat (PCai1, established from a TRAP prostate tumor) and human (22Rv1) CRPC cells	miR-8080 mediated suppression of AR-V7	[68]

## Conclusion

Nature-derived polyphenolics have been long known for their medicinal competence and therapeutic precedence with minimal side effects and toxicity. The physiological benevolence of natural chemicals such as luteolin has been attracted considerable attention for its application in the mitigation of deadly diseases. Luteolin targets multifaceted cancer pathways by creating redox stress, ROS generation, cell cycle arrest, inducing autophagy, onset of apoptosis, inhibition of cell proliferation, eventually leading to cancer cell death. Luteolin is known to exert synergistic inhibition of cancer progression with anticancer drugs, and its anticancer profile is successfully applied for the management of colon cancer, breast cancer, prostate cancer, and liver cancer. The parent structure of luteolin has been chemically modified to further achieve enhanced bioavailability for an optimal therapeutic efficacy. However, the mechanism of inhibition of cancer progression by luteolin is still not well elucidated. The untangling of luteolin-mediated anticancer effect can serve as a promising approach for the development of luteolin as the anticancer drug of future. Similarly, luteolin is safer however some reports have indicated the worsening of chemically induced colitis in animal models that raises the necessity address the safety profile of luteolin.

## Data Availability

Not applicable.
